# Association between Urinary Creatinine Excretion and Hypothyroidism in Patients with Chronic Kidney Disease

**DOI:** 10.3390/diagnostics13040669

**Published:** 2023-02-10

**Authors:** Natsumi Matsuoka-Uchiyama, Kenji Tsuji, Kensaku Takahashi, Kazuhiko Fukushima, Hidemi Takeuchi, Shinji Kitamura, Kenichi Inagaki, Haruhito A. Uchida, Jun Wada

**Affiliations:** 1 Department of Nephrology, Rheumatology, Endocrinology and Metabolism, Okayama University Graduate School of Medicine, Dentistry and Pharmaceutical Sciences, 2-5-1 Shikata-cho, Okayama 700-8558, Japan; 2 Department of Chronic Kidney Disease and Cardiovascular Disease, Okayama University Graduate School of Medicine, Dentistry and Pharmaceutical Sciences, 2-5-1 Shikata-cho, Okayama 700-8558, Japan

**Keywords:** hypothyroidism, kidney function, urinary creatinine excretion

## Abstract

While hypothyroidism increases serum creatinine (Cr) levels, it is uncertain whether the elevation is mediated via a decline in the glomerular filtration rate (GFR) or the reflection of enhanced Cr production from the muscles or both. In the present study, we explored an association between urinary Cr excretion rate (CER) and hypothyroidism. A total of 553 patients with chronic kidney disease were enrolled in a cross-sectional study. Multiple linear regression analysis was performed to explore the association between hypothyroidism and urinary CER. The mean urinary CER was 1.01 ± 0.38 g/day and 121 patients (22%) had hypothyroidism. The multiple linear regression analysis revealed explanatory variables with urinary CER, including age, sex, body mass index, 24 h Cr clearance (24hrCcr), and albumin while hypothyroidism was not considered an independent explanatory variable. In addition, scatter plot analysis with regression fit line representing the association between estimated GFR calculated using s-Cr (eGFRcre) and 24hrCcr revealed that eGFRcre and 24hrCcr had strong correlations with each other in hypothyroid patients as well as euthyroid patients. Collectively, hypothyroidism was not considered an independent explanatory variable for urinary CER in the present study and eGFRcre is a useful marker to evaluate kidney function regardless of the presence of hypothyroidism.

## 1. Introduction

It is well known that there is a strong interaction between thyroid function and kidney function [1]. An elevation in serum creatinine (s-Cr) levels is reported to be seen in hypothyroidism [2], and the replacement of thyroid hormone reversed the elevation of s-Cr levels. Similarly, a decline in s-Cr levels is observed in hyperthyroidism, and reversal of s-Cr can be seen after the normalization of thyroid function [3], suggesting that thyroid function affects s-Cr levels. While details of the mechanisms are still uncertain, it is suggested that hypothyroidism may cause renal dysfunction by the following mechanisms: reduced renal perfusion pressure through the decrease in cardiac output and the increase in vascular resistance [4,5], reduced sensitivity to the body’s sympathetic drive and renin–angiotensin–aldosterone system activity [6], and possible rhabdomyolysis [7].

However, it is still controversial whether thyroid function really regulates the glomerular filtration rate (GFR). While previous reports indicated decreased creatinine clearance in hypothyroidism [8], other reports suggested that increased s-Cr levels under hypothyroidism might be just the reflection of increased creatinine (Cr) production from the muscles independent of the GFR [9,10]. These mechanisms may include the following increased processes such as (1) synthesis of creatine, (2) storage of creatine in the muscle, (3) creatine–phosphocreatine cycle, (4) conversion of creatine or phosphocreatine into Cr, and (5) release of Cr from muscle tissue due to myopathy and/or rhabdomyolysis [9,10]. Indeed, serum levels of muscle enzymes including creatine phosphokinase have been reported to be increased in hypothyroidism [11]. What happens if hypothyroidism increases Cr production? Since the Cr production should equal urinary Cr excretion when the s-Cr levels are in a steady state [12], the increase in Cr production results in the increase in s-Cr levels independent of the GFR. In this situation, the estimated GFR calculated using s-Cr (eGFRcre) may not be a reliable measurement for kidney function. In contrast, 24 h Cr clearance (24hrCcr) levels are not affected by Cr production levels. Cystatin C (CysC), another marker of kidney function, has advantages for patients with muscle weakness since the evaluation is independent of muscle mass [13]. Since hypothyroidism reduces CysC production through a metabolic-rate-mediated mechanism [14] and treatment for hypothyroidism affects serum CysC levels [15], CysC is proposed as a possible biomarker for monitoring hypothyroidisms [16].

Since most of the previous reports analyzed the association between hypothyroidism and kidney function by eGFRcre [17,18,19], it is currently uncertain how hypothyroidism increases s-Cr levels, via an increase in urinary Cr excretion rate (CER) or a decrease in GFR, or both. In the present study, we conducted a cross-sectional study to evaluate the association between hypothyroidism and urinary CER.

## 2. Materials and Methods

### 2.1. Study Design and Participants

**Figure 1:**
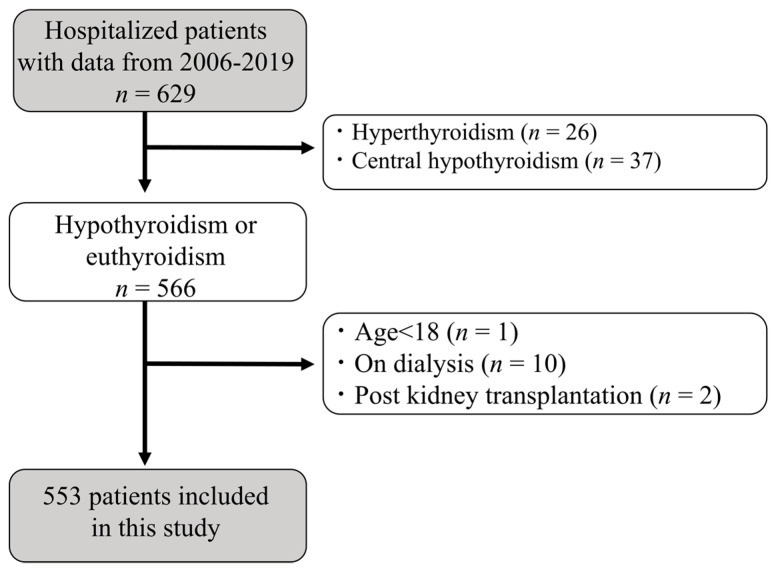
Flow diagram of the screening and enrollment of study patients.

We retrospectively reviewed patients with a measurement of 24 h urine collection while hospitalized in the division of kidney, diabetes, and endocrine diseases at Okayama University Hospital. Data from 2006 to 2019 were collected from electronic-based records. Data collection was completed in 2020–2021. Our database of medical records provided 18,068 patients. Among these patients, we collected 629 patients with all the following data of the measurements of s-Cr, 24hrCcr, thyroid-stimulating hormone (TSH), free thyroxine (FT4), free triiodothyronine (FT3), 24 h urinary protein and Cr, total cholesterol, serum albumin, CysC, and glycated hemoglobin (HbA1c). We extracted 566 hypothyroid and euthyroid patients after excluding hyperthyroid and central hypothyroid patients. Other exclusion criteria were as follows: (1) age <18 years, (2) on dialysis, and (3) post-kidney transplantation. Consequently, 553 patients with chronic kidney disease were enrolled for a cross-sectional analysis (shown in Figure 1). The study was approved by the ethics committees of Okayama University Hospital Institutional Review Board (accredited ISO9001/2000), approved number (OKU-206-022). This study also followed the Declaration of Helsinki on medical protocol and ethics. As for informed consent, the contents of the research were posted on our department homepage and the hospital, and public informed consent was provided. As this is a cross-sectional study, the committee approved public informed consent.

### 2.2. Data Collection

The following clinicopathologic characteristics were collected at the time of the hospitalization: age, sex, body mass index (BMI), and the use of glucocorticoid, levothyroxine, angiotensin-converting-enzyme inhibitor (ACE-i), and angiotensin II receptor blocker (ARB). HbA1c data are presented as National Glycohemoglobin Standardization Program values according to the recommendations of the Japanese Diabetes Society and International Federation of Clinical Chemistry [20]. eGFRcre was evaluated by the equation developed by the Japanese Society of Nephrology (eGFRcre (mL/min/1.73 m^2^) = 194 × s-Cr (mg/dL)^−1.094^ × Age^−0.287^ (× 0.739 for females)) [21]. While the Chronic Kidney Disease Epidemiology Collaboration (CKD-EPI) equation [22] or the Modification of Diet in Renal Disease (MDRD) study equation is applied for eGFR calculation worldwide [23], it is reported that these equations tend to overestimate Japanese kidney function [24,25]. Since all the patients enrolled were Japanese, we applied the modified equation for Japanese in the study, which is widely used in Japan [21]. Nephrotic syndrome (NS) was defined by both substantial proteinuria (>3.5 g/24 h) and hypoalbuminemia (<3.0 g/dL). The 24hrCcr was calculated by: 24hrCcr (mL/min) = 24 h urinary Cr (mg/dL) × 24 h collected urine (mL/day)/24 (hour)/60 (min)/s-Cr (mg/dL) × 1.73/BSA. BSA was calculated by: BSA (m^2^) = body weight^0.425^ × height^0.725^ × 0.007184 [26]. Participants collected 24 h urine accurately, supervised by nurses in the hospital. Urine volume was recorded (mL), and Cr was measured by the enzyme method. Urinary CER was calculated by: urinary CER (g/day) = urine volume (mL/day) x urinary Cr (mg/dL)/10^5^. Thyroid function was measured by using an electrochemiluminescence assay (Roche Diagnostics K.K., Cobas^®^ 8000). The normal reference range in our institute for TSH was 0.27–4.2 µIU/mL, FT4 was 0.93–1.7 ng/dL, and FT3 was 2.3–4.0 pg/mL. In this study, hypothyroidism was defined as combined overt hypothyroidism (FT4 < 0.93 ng/dL and TSH > 4.2 µIU/L) and subclinical hypothyroidism (0.93 ≤ FT4 ≤ 1.7 ng/dL and TSH > 4.2 µIU/mL) like the previous reports [27,28]. Euthyroidism was defined as the status with normal TSH and FT4 regardless of their current or history of thyroidal diseases.

### 2.3. Statistical Analysis

The statistical analyses were performed by JMP version 14.0.0 (SAS Institute, Inc, Cary, NC, USA) and Stata/SE version 16.1 (Stata Corp LLC, College Station, TX, USA). Significance was defined as *p*-values less than 0.05. Data were expressed as *n* (%) for categorical variables and mean ± standard deviation (SD) for continuous variables. Categorical variables were analyzed with the chi-square test, while continuous variables were compared by using the student’s *t*-test or Mann–Whitney U test as appropriate. Correlations among urinary CER and continuous variables (age, eGFRcre, BMI, 24hrCcr, FT4, TSH, and FT3) were evaluated by Spearman’s correlation analysis. Multiple linear regression analysis was performed to explore the association of variates with urinary CER. For the analysis, candidate variables contained hypothyroidism, sex, age, BMI, and 24hrCcr. Correlations among 24hrCcr and eGFRcre under euthyroidism and hypothyroidism were evaluated by Spearman’s correlation analysis. *p* for trend was calculated by the Cochran–Armitage trend test or the Jonckheere–Terpstra test.

## 3. Results

### 3.1. Study Population and Clinical Characteristics

Among 629 patients in all the data sets, 553 patients met the selection criteria and were enrolled for the analysis (Figure 1). There were no patients with acute kidney injury or rhabdomyolysis. The characteristics of the participants stratified by tertiles of urinary CER are shown in Table 1. The average age was 60 ± 15 years and 52% of the patients were men. The average level of eGFRcre and 24hrCcr was 55.7 ± 30.5 mL/min/1.73 m^2^ and 64.6 ± 38.4 mL/min, respectively. The average urinary CER was 1.01 ± 0.38 g/day. Trend analyses revealed that patients showed higher 24hCCr, eGFRcre, albumin, hemoglobin, and FT3 levels (all the *p*-value for trends <0.001), younger age (*p*-value for trends <0.001), higher proportion of male gender (*p*-value for trends <0.001), higher BMI (*p*-value for trends <0.001), and lower proportion of hypothyroidism (*p*-value for trends 0.016) with higher urinary CER. To analyze the association between hypothyroidism and urinary CER, we stratified the patients by the presence or absence of hypothyroidism (Table 2). The number of participants with hypothyroidism and euthyroidism were 121 and 432, respectively. Patients with hypothyroidism had higher levels of s-Cr and urinary protein, had NS more frequently, and received more ACE-i/ARB agents. Patients with hypothyroidism also had lower eGFRcre, 24hrCcr, urinary CER, serum albumin, and HbA1c levels.

### 3.2. Hypothyroidism Is Not an Independent Explanatory Variable for Urinary CER

**Figure 2:**
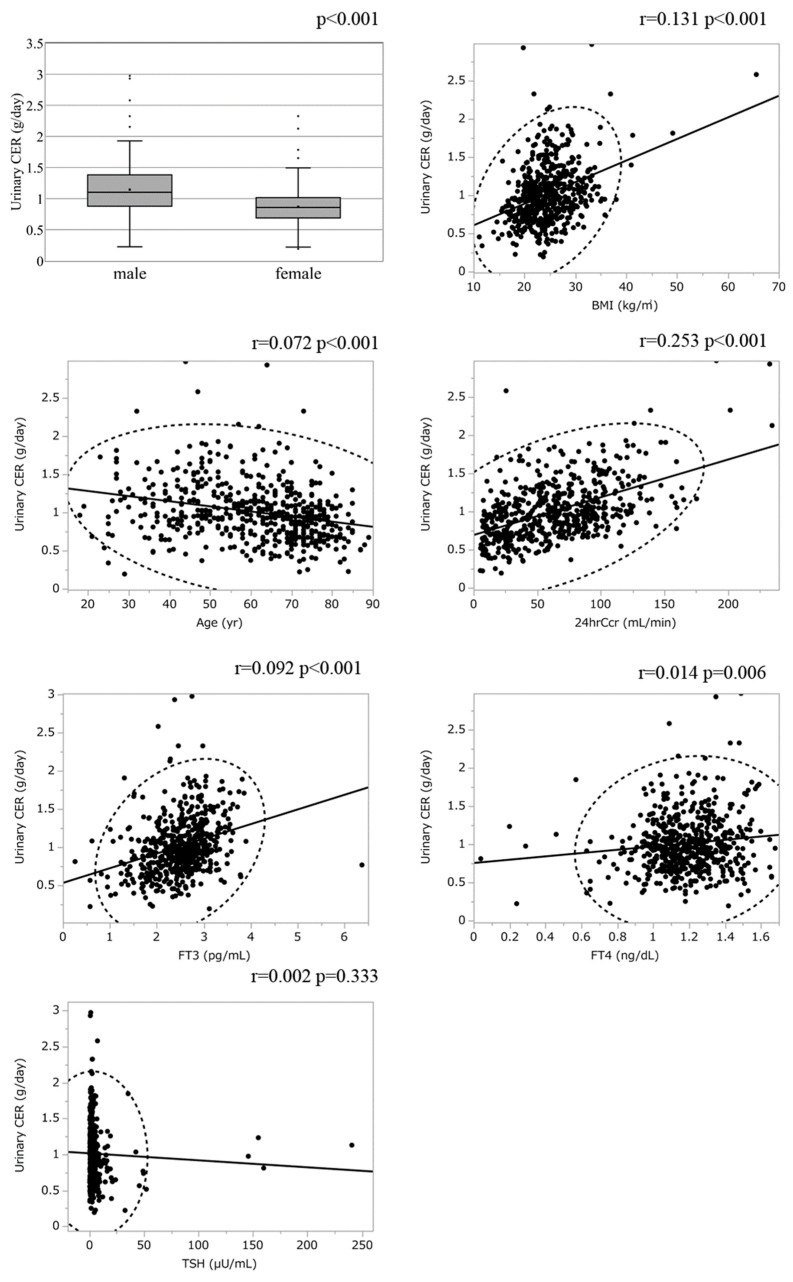
The association between urinary creatinine excretion rate and variables. Urinary CER, urinary creatinine excretion rate; eGFRcre, estimated glomerular filtration rate calculated by serum creatinine; BMI, body mass index; 24hrCcr, 24 h creatinine clearance; FT4, free thyroxine; TSH, thyroid-stimulating hormone; FT3, free triiodothyronine.

**Figure 3:**
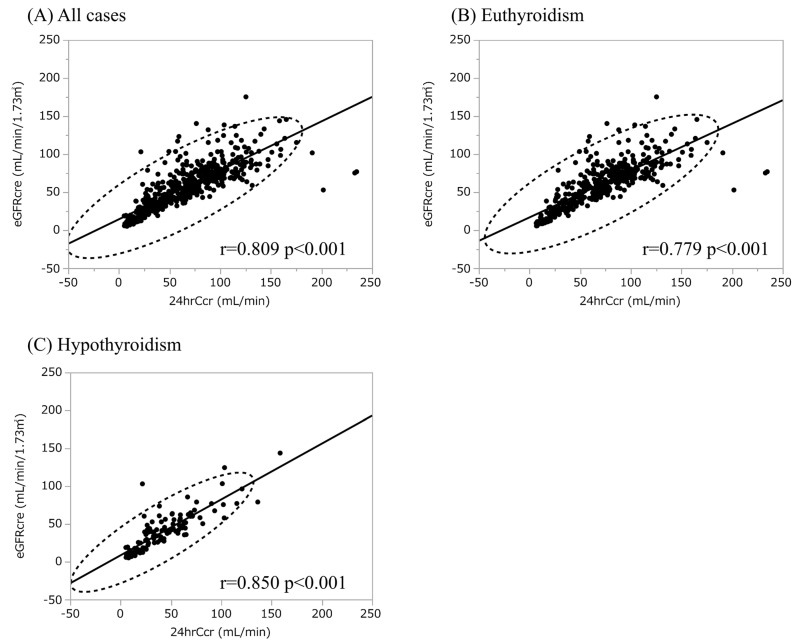
The association between eGFRcre and 24hCcr. (**A**) The association between eGFRcre and 24hCcr in all cases. (**B**) The association between eGFRcre and 24hCcr in euthyroid cases. (**C**) The association between eGFRcre and 24hCcr in total hypothyroid cases. eGFRcre, estimated glomerular filtration rate calculated by serum creatinine; 24hrCcr, 24 h creatinine clearance.

To explore whether thyroid function affects Cr production, we compared the urinary CER between euthyroidism and hypothyroidism (Table 2). The urinary CER was rather lower in hypothyroid patients compared with the levels in euthyroid patients. The enrolled patients with hypothyroidism showed lower kidney function and protein-energy wasting syndrome, i.e., lower skeletal muscle mass, which may contribute to reduced urinary CER [29,30,31]. We next conducted scatter plot analysis to explore the association between urinary CER with variables, which revealed a weak positive correlation with 24hrCcr while there was no apparent correlation with age, BMI, FT3, FT4, and TSH (Figure 2). Male patients had higher urinary CER compared with female patients, compatible with the previous report (Figure 2). To further analyze the explanatory variables for urinary CER, univariate and multiple linear regression analyses were applied (Table 3 and Table 4), in which age, sex, BMI 24hrCcr, and albumin were detected as an independent explanatory variable for urinary CER while neither hypothyroidism nor FT3 was detected. Because both thyroid function and renal function are affected by NS [32], we also conducted the analyses using the data excluding patients with NS. The characteristics of the participants without NS stratified by thyroid function and the results of multiple linear regression analyses are shown in the Supplementary Materials, Tables S1 and S2, respectively, in which neither hypothyroidism nor FT3 was detected as an independent explanatory variable for urinary CER. Collectively, it is unlikely that an increase in s-Cr is also mediated by increased Cr production due to hypothyroidism, suggesting that eGFRcre is a reliable marker to evaluate kidney function even under hypothyroidism. To further evaluate the reliability of eGFRcre under hypothyroidism, we conducted scatter plot analysis with a regression fit line representing the association between eGFRcre and 24hrCcr under the categories of thyroid function (Figure 3), which revealed that eGFR and 24hrCcr had strong correlations with each other in hypothyroid patients as well as euthyroid patients (r = 0.850; *p* < 0.001 and r = 0.779; *p* < 0.001, respectively).

## 4. Discussion

**Figure 4:**
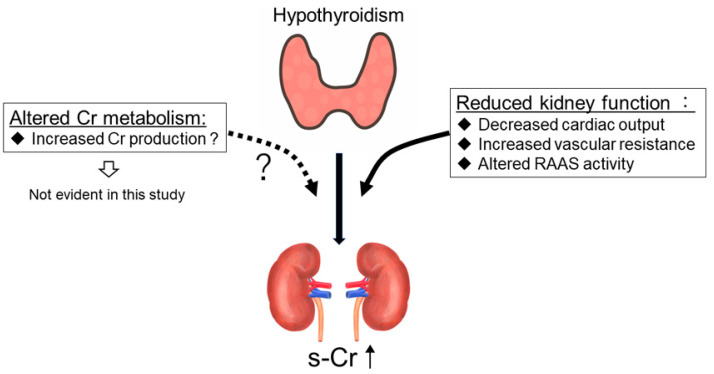
The mechanisms influencing serum creatinine levels under hypothyroidism. Cr, creatinine; s-Cr, serum creatinine; RAAS, renin–angiotensin–aldosterone system.

While it is well known that hypothyroidism increases s-Cr levels [3], details of the mechanisms are still uncertain. It is suggested that hypothyroidism reduces kidney function via decreased cardiac output, increased vascular resistance, and an altered renin–angiotensin–aldosterone system [4,5,6] (Figure 4). In addition, it has also been suggested that increased s-Cr levels under hypothyroidism are via altered Cr metabolism by increased Cr production [9] (Figure 4). In the present study, hypothyroidism was not detected as an explanatory variable for urinary CER. Thus, it is unlikely that hypothyroidism increases Cr production (Figure 4), suggesting that increased s-Cr levels under hypothyroidism are predominantly via reduced kidney function. It is estimated that more than 98% of Cr is from muscle [33], where it is produced and secreted into serum at a continuous rate [34]. S-Cr is almost exclusively excreted in the urine [35]. Thus, the urinary CER, reported to be around 1 g/day [36], has been recognized as a marker of muscle mass [37], and Cr generation must approximately equal Cr excretion under stable kidney function. Indeed, it is reported that the large variance in urinary CER was accounted for by muscle mass determined by dual energy x-ray absorptiometry, and mid-arm muscle area by computed tomography [12,38,39]. In general, muscle mass does not change rapidly within individuals, and elevations in s-Cr typically reflect decrements in GFR. Under hypothyroidisms, several muscle manifestations may be observed including muscle weakness, myalgia, muscle cramps, and Hoffman syndrome as well as rhabdomyolysis [7]. Though the pathogenesis of myopathy caused by hypothyroidisms is not entirely understood, several mechanisms, such as altered glycogenolytic and oxidative metabolism, contractile proteins, and neuro-mediated damages, were proposed [7]. While muscle damages may affect the Cr generation and/or secretion from the muscles, which may result in the increase in urinary CER, we did not find a significance regarding urinary CER between hypothyroidisms and euthyroidisms. Nevertheless, hypothyroidisms-induced rhabdomyolysis need to be carefully monitored since rhabdomyolysis may cause acute kidney injury [40]. Similar to muscle mass, urinary CER is reported to have a positive correlation with male gender, and a negative correlation with age and eGFRcre [12,41,42]. Lower urinary CER is reported to be associated with death and end-stage renal disease among chronic kidney disease (CKD) patients, indicating the clinical significance of urinary CER [42].

While the presence of hypothyroidisms was not an independent explanatory variable for urinary CER, age, sex, BMI, and 24hrCcr were associated with urinary CER as previously reported [12,41,42]. In addition, albumin was also detected as an independent explanatory variable for urinary CER. Patients of younger age, higher BMI, and higher 24hrCcr as well as male gender showed higher urinary CER probably because these patients are more likely to have more muscle mass. We also indicated that eGFRcre and 24hrCcr had strong correlations with each other under hypothyroidism as well as under euthyroidism, implying the parallel fluctuation between 24hrCcr and eGFRcre. Collectively, eGFRcre is a reliable marker to evaluate kidney function regardless of the presence or absence of hypothyroidism. Since it has been reported that thyroid hormone replacement therapy significantly improved kidney function and attenuated the rate of decline in kidney function in CKD patients with hypothyroidism [43], the appropriate treatment with the appropriate diagnosis of hypothyroidism may delay CKD progression. 

There are several limitations in this study. First, the sample size is relatively small in a single university hospital and the participants are mainly hospitalized for education or examination in the division of kidney, diabetes, and endocrine diseases, thus most of the participants had CKD and diabetes mellitus; therefore, selection bias cannot be excluded. In addition, kidney and thyroid function might be affected due to the medical condition for hospitalization. Therefore, it is difficult to generalize our results to patients without CKD and diabetes mellitus. Second, 52 patients with NS were included in the study. Since hypothyroidism is a well-known complication of NS [32] due to the increased urinary excretion of thyroid hormone and thyroxine-binding globulin [44], the degree of proteinuria may affect the results. Nevertheless, the univariate regression analysis revealed that the level of urinary protein excretion was not an explanatory variable for urinary CER. In addition, we also conducted the multivariate analysis using the data excluding patients with NS, in which hypothyroidism was not detected as an independent explanatory variable for urinary CER, suggesting that the inclusion of patients with NS is less likely to affect the conclusion. Third, thyroid autoantibodies or other autoimmune antibodies were not evaluated in this study. Therefore, the possibility of a common underlying autoimmune process related to hypothyroidism cannot be excluded. Forth, we were unable to exclude the possible effect on Cr secretion into renal tubules under thyroid dysfunction. To exclude this possibility, further analysis, such as a comparison with inulin clearance or 51Cr-EDTA clearance [45] as an accurate method to measure GFR is required. Lastly, a cross-sectional study is insufficient to draw definitive conclusions about the association between hypothyroidism and urinary CER; therefore, future prospective analyses with measurements of urinary CER, renal function, and thyroid function as well as muscle mass before and after thyroid hormone replacement therapy are required for further clarification.

## 5. Conclusions

Our study indicated that hypothyroidism was not considered an independent explanatory variable for urinary CER in the present study and eGFRcre is reliable to evaluate kidney function regardless of the presence of hypothyroidism. Future prospective studies are required for further clarification.

## Figures and Tables

**Table 1:** Characteristics of study participants stratified by tertiles of urinary creatinine excretion.

Clinical Parameters	All Participants (*n* = 553)	Low CER (*n* = 184)	Middle CER (*n* = 184)	High CER (*n* = 185)	*p* for Trend
Sex (Male), *n* (%)	285 (52)	61 (33)	83 (45)	141 (76)	<0.001 **
Age (yr)	60 ± 15	65 ± 15	61 ± 14	55 ± 14	<0.001 **
BMI (kg/m^2^)	24.3 ± 4.8	22.3 ± 3.9	24.6 ± 4.3	26.1 ± 5.4	<0.001 **
TSH (µU/mL)	4.88 ± 15.97	5.54 ± 13.99	4.29 ± 11.33	4.81 ± 21.03	0.004 **
FT4 (ng/dL)	1.19 ± 0.21	1.17 ± 0.22	1.17 ± 0.19	1.22 ± 0.20	0.052
FT3 (pg/mL)	2.49 ± 0.60	2.27 ± 0.67	2.50 ± 0.49	2.72 ± 0.53	<0.001 **
Hypothyroidism *n* (%)	121 (22)	48 (26)	44 (24)	29 (16)	0.016 *
s-Cr (mg/dL)	1.44 ± 1.27	1.62 ± 1.41	1.44 ± 1.37	1.26 ± 0.97	0.826
eGFRcre (mL/min/1.73 m^2^)	55.7 ± 30.5	51.5 ± 35.8	55.2 ± 27.7	60.4 ± 26.9	<0.001 **
24hrCcr (mL/min)	64.6 ± 38.4	45.3 ± 31.1	64.9 ± 32.8	83.7 ± 40.7	<0.001 **
Urinary output (mL/day)	1665 ± 710	1447 ± 617	1674 ± 674	1873 ± 769	<0.001 **
Urinary CER (g/day)	1.01 ± 0.38	0.64 ± 0.14	0.97 ± 0.07	1.42 ± 0.31	<0.001 **
Urinary protein (g/day)	2.1 ± 6.7	1.7 ± 3.9	2.5 ± 9.0	2.3 ± 6.3	0.562
Albumin (g/dL)	3.6 ± 0.8	3.4 ± 0.8	3.6 ± 0.8	3.8 ± 0.8	<0.001 **
Hemoglobin (g/dL)	12.3 ± 2.3	11.6 ± 2.3	12.2 ± 2.1	13.2 ± 2.4	<0.001 **
Total cholesterol (mg/dL)	196 ± 62	192 ± 63	196 ± 62	200 ± 60	0.134
HbA1c (%)	7.1 ± 2.0	6.9 ± 2.0	7.2 ± 2.1	7.1 ± 2.1	0.370
Nephrotic syndrome *n* (%)	52 (9)	19 (10)	20 (11)	13 (7)	0.277
ACE-i/ARB intake *n* (%)	128 (23)	40 (22)	42 (23)	46 (25)	0.477
Levothyroxine intake *n* (%)	55 (10)	23 (13)	18 (10)	14 (8)	0.114
Glucocorticoid intake *n* (%)	26 (5)	13 (7)	5 (3)	8 (4)	0.215

BMI, body mass index; TSH, thyroid-stimulating hormone; FT4, free thyroxine; FT3, free triiodothyronine; s-Cr, serum creatinine; eGFRcre, estimated glomerular filtration rate calculated by serum creatinine; 24hrCcr, 24 h creatinine clearance; urinary CER, urinary creatinine excretion rate; HbA1c, glycated hemoglobin; ACE-i, angiotensin-converting-enzyme inhibitor; ARB, angiotensin II receptor blocker. *p* for trend was calculated by the Cochran–Armitage trend test or the Jonckheere–Terpstra test. * *p* < 0.05, ** *p* < 0.01.

**Table 2:** Characteristics of study participants stratified by thyroidal status.

Clinical Parameters	All Participants (*n* = 553)	Hypothyroidism (*n* = 121)	Euthyroidism (*n* = 432)	*p*-Value
Sex (Male), *n* (%)	285 (52)	66 (55)	219 (51)	0.454
Age (yr)	60 ± 15	63 ± 16	59 ± 15	0.224
BMI (kg/m2)	24.3 ± 4.8	24.8 ± 6.0	24.2 ± 4.8	0.208
TSH (µIU/mL)	4.88 ± 15.97	15.74 ± 31.91	1.84 ± 15.97	<0.001 **
FT4 (ng/dL)	1.19 ± 0.21	1.03 ± 0.26	1.23 ± 0.16	<0.001 **
FT3 (pg/mL)	2.49 ± 0.60	2.25 ± 0.73	2.56 ± 0.53	<0.001 **
s-Cr (mg/dL)	1.44 ± 1.27	2.14 ± 1.67	1.24 ± 1.06	<0.001 **
Cystatin C (mg/L)	1.66 ± 1.06	2.21 ± 1.17	1.51 ± 0.98	<0.001 **
eGFRcre (mL/min/1.73 m^2^)	55.7 ± 30.5	38.9 ± 26.0	60.5 ± 30.1	<0.001 **
24hrCcr (mL/min)	64.6 ± 38.4	41.4 ± 29.9	71.2 ± 38.0	<0.001 **
Urinary output (mL/day)	1665 ± 710	1579 ± 745	1689 ± 699	0.134
Urinary CER (g/day)	1.01 ± 0.38	0.91 ± 0.35	1.04 ± 0.38	0.001 **
Urinary protein (g/day)	2.1 ± 6.7	3.5 ± 5.7	1.8 ± 7.0	<0.001 **
Albumin (g/dL)	3.6 ± 0.8	3.2 ± 1.0	3.7 ± 0.7	<0.001 **
Hemoglobin (g/dL)	12.3 ± 2.3	12.0 ± 2.4	12.5 ± 2.3	0.042 *
Total cholesterol (mg/dL)	196 ± 62	204 ± 72	194 ± 59	0.110
HbA1c (%)	7.1 ± 2.0	6.5 ± 1.6	7.2 ± 2.1	<0.001 **
Nephrotic syndrome *n* (%)	52 (9)	25 (21)	27 (6)	<0.001 **
ACE-i/ARB intake *n* (%)	128 (23)	40 (33)	88 (20)	0.003 **
Levothyroxine intake *n* (%)	55 (10)	34 (28)	21 (5)	<0.001 **
Glucocorticoid intake *n* (%)	26 (5)	7 (6)	19 (4)	0.524

BMI, body mass index; TSH, thyroid-stimulating hormone; FT4, free thyroxine; FT3, free triiodothyronine; s-Cr, serum creatinine; eGFRcre, estimated glomerular filtration rate calculated by serum creatinine; 24hrCcr, 24 h creatinine clearance; urinary CER, urinary creatinine excretion rate; HbA1c, glycated hemoglobin; ACE-i, angiotensin-converting-enzyme inhibitor; ARB, angiotensin II receptor blocker. *p*-values were obtained by Student’s *t*-test or Mann–Whitney U test or Pearson’s chi-square test. * *p* < 0.05, ** *p* < 0.01.

**Table 3:** Univariate regression analysis of urinary creatinine excretion rate and variables.

Variable	B	95% CI	β	t	*p*-Value
Sex (Male)	0.138	0.108 to 0.167	0.366	9.22	<0.001 **
Age	−0.007	−0.009 to −0.005	−0.267	−6.52	<0.001 **
BMI	0.028	0.022 to 0.034	0.362	9.12	<0.001 **
TSH	−0.001	−0.003 to 0.001	−0.041	−0.97	0.333
FT4	0.217	0.064 to 0.369	0.118	2.79	0.006 **
FT3	0.192	0.142 to 0.243	0.304	7.48	<0.001 **
Hypothyroidism	0.064	0.027 to 0.103	0.143	3.39	<0.001 **
s-Cr	−0.034	−0.058 to −0.009	−0.113	−2.67	0.008 **
eGFRcre	0.002	0.001 to 0.003	0.135	3.21	0.001 **
24hrCcr	0.005	0.004 to 0.006	0.503	13.65	<0.001 **
Urinary output	0.000	0.000 to 0.000	0.271	6.60	<0.001 **
Urinary protein	0.001	−0.003 to 0.006	0.026	0.62	0.536
Albumin	0.099	0.061 to 0.137	0.212	5.09	<0.001 **
Hemoglobin	0.045	0.032 to 0.058	0.277	6.76	<0.001 **
Total cholesterol	0.000	−0.000 to 0.001	0.015	0.36	0.719
HbA1c	0.018	0.002 to 0.033	0.096	2.27	0.024 *
Nephrotic syndrome	−0.031	−0.085 to 0.023	−0.048	−1.14	0.255
ACE-i/ARB intake	0.005	0.032 to 0.043	0.012	0.28	0.779
Levothyroxine intake	−0.052	−0.105 to −0.000	−0.083	−1.97	0.050 *
Glucocorticoid intake	0.064	−0.010 to 0.138	0.072	1.69	0.091

BMI, body mass index; TSH, thyroid-stimulating hormone; FT4, free thyroxine; FT3, free triiodothyronine; s-Cr, serum creatinine; eGFRcre, estimated glomerular filtration rate calculated by serum creatinine; 24hrCcr, 24 h creatinine clearance; urinary CER, urinary creatinine excretion rate; HbA1c, glycated hemoglobin; ACE-i, angiotensin-converting-enzyme inhibitor; ARB, angiotensin II receptor blocker; B, unstandardized regression coefficient; β, standardized coefficient; CI, confidence interval for B. * *p* <0.05, ** *p* < 0.01.

**Table 4:** Multiple regression analysis of urinary creatinine excretion rate and variables. (**A**) Adjusted for presence of hypothyroidism, sex, age, BMI, 24hCcr, albumin, and hemoglobin. (**B**) Adjusted for FT3, sex, age, BMI, 24hCcr, albumin, and hemoglobin.

Variable	B	95% CI	β	t	*p*-Value
(**A**)					
Hypothyroidism	0.025	−0.035 to 0.085	0.031	0.83	0.407
Sex (male)	0.173	0.152 to 0.194	0.459	16.23	<0.001 **
Age	−0.003	−0.004 to −0.001	−0.107	−3.60	<0.001 **
BMI	0.027	0.023 to 0.031	0.344	12.49	<0.001 **
24hrCcr	0.005	0.005 to 0.006	0.526	15.95	<0.001 **
Albumin	0.050	0.023 to 0.078	0.108	3.61	<0.001 **
Hemoglobin	−0.004	−0.014 to 0.005	−0.027	−0.89	0.372
Constant	0.067				<0.001 **
(**B**)					
FT3	0.017	−0.024 to 0.058	0.027	0.82	0.410
Sex (male)	0.172	0.151 to 0.192	0.455	16.05	<0.001 **
Age	−0.003	−0.004 to −0.001	−0.105	−3.54	<0.001 **
BMI	0.027	0.022 to 0.031	0.342	12.36	<0.001 **
24hrCcr	0.005	0.004 to 0.006	0.512	16.02	<0.001 **
Albumin	0.043	0.013 to 0.072	0.091	2.87	0.004 **
Hemoglobin	−0.004	−0.014 to 0.005	−0.028	−0.90	0.368
Constant	0.049				<0.001 **

BMI, body mass index; 24hrCcr, 24 h creatinine clearance; B, unstandardized regression coefficient; ꞵ, standardized coefficient; CI, confidence interval for B. Model R^2^ = 0.596, adjusted R^2^ = 0.590. **p < 0.01. FT3, free triiodothyronine; BMI, body mass index; 24hrCcr, 24 h creatinine clearance; B, unstandardized regression coefficient; ꞵ, standardized coefficient; CI, confidence interval for B. Model R2 = 0.595, adjusted R2 = 0.590. ** *p* < 0.01.

## Data Availability

The datasets generated during and/or analyzed during the current study are not publicly available as these have not been anonymized but are available from the corresponding author on reasonable request.

## Supplementary Materials

The following supporting information can be downloaded at: https://www.mdpi.com/article/10.3390/diagnostics13040669/s1, Table S1: Characteristics of study participants without nephrotic syndrome stratified by thyroidal status; Table S2: Multiple regression analysis of urinary creatinine excretion rate and variables in patients without nephrotic syndrome.
