# Supplementary material for: Association between Urinary Creatinine Excretion and Hypothyroidism in Patients with Chronic Kidney Disease
Source: Diagnostics (Basel). 2023 Feb 10;13(4):669. doi: 10.3390/diagnostics13040669 (PMC9955896; doi:10.3390/diagnostics13040669)
Supplement: Supplementary file 1 [file diagnostics-13-00669-s001.zip › diagnostics-2184642-supplementary.docx]

**Supplementary Table S1. Characteristics of study participants without nephrotic syndrome stratified by thyroidal status.**

| Clinical Parameters | all participants  (n = 501) | hypothyroidism  (n = 96) | euthyroidism  (n =432) | *P-*value |
| --- | --- | --- | --- | --- |
| Sex (Male), n (%) | 285 (52) | 49 (51) | 203 (81) | 0.872 |
| Age (yr) | 60±15 | 63±15 | 59±15 | 0.012^*^ |
| BMI (kg/m^2^) | 24.3±4.8 | 24.1±4.8 | 24.2±4.5 | 0.541 |
| TSH (µU/mL) | 4.88±15.97 | 17.57±35.56 | 1.82±16.71 | <0.001^**^ |
| FT4 (ng/dL) | 1.19±0.21 | 1.03±0.28 | 1.24±0.16 | <0.001^**^ |
| FT3 (pg/mL) | 2.49±0.60 | 2.30±0.74 | 2.59±0.52 | <0.001^**^ |
| s-Cr (mg/dL) | 1.44±1.27 | 2.19±1.70 | 1.20±1.01 | <0.001^**^ |
| Cystatin C (mg/L) | 1.66±1.06 | 2.25±1.21 | 1.46±0.95 | <0.001^**^ |
| eGFRcre (mL/min/1.73m^2^) | 55.7±30.5 | 38.3±27.4 | 61.5±29.7 | <0.001^**^ |
| 24hrCcr (mL/min) | 64.6±38.4 | 41.9±32.0 | 72.6±37.8 | <0.001^**^ |
| Urinary output (mL/day) | 1665±710 | 1595±672 | 1671±684 | 0.277 |
| Urinary CER (g/day) | 1.01±0.38 | 0.90±0.33 | 1.04±0.39 | 0.002^**^ |
| Urinary protein (g/day) | 2.1±6.7 | 1.5±2.0 | 0.7±1.4 | <0.001^**^ |
| Albumin (g/dL) | 3.6±0.8 | 3.6±0.7 | 3.8±0.6 | 0.003^**^ |
| Hemoglobin (g/dL) | 12.3±2.3 | 11.9±2.4 | 12.5±2.3 | 0.013^*^ |
| Total cholesterol (mg/dL) | 196±62 | 183±49 | 190±53 | 0.292 |
| HbA1c (%) | 7.1±2.0 | 6.5±1.5 | 7.3±2.1 | <0.001^**^ |
| ACE-i/ARB intake n (%) | 128 (23) | 32 (33) | 77 (19) | 0.002^**^ |
| Levothyroxine intake n (%) | 128 (23) | 28 (29) | 19 (5) | <0.001^**^ |
| Glucocorticoid intake n (%) | 128 (23) | 4 (4) | 18 (4) | 0.905 |

BMI, body mass index; TSH, thyroid-stimulating hormone; FT4, free thyroxine; FT3, free triiodothyronine; s-Cr, serum creatinine; eGFRcre, estimated glomerular filtration rate calculated by serum creatinine; 24hrCcr, 24-hour **creatinine** clearance; urinary CER, urinary creatinine excretion rate; HbA1c, glycated hemoglobin; ACE-i, angiotensin-converting-enzyme inhibitor; ARB, angiotensin II receptor blocker. *P-*values were obtained by Student’s t-test or Mann–Whitney U test or Pearson’s chi-square test. *P <0.05, **P<0.01.

**Supplementary Table S2. Multiple regression analysis of urinary creatinine excretion rate and variables in patients without nephrotic syndrome.**

| **I: Adjusted for presence of hypothyroidism, sex, age, BMI, 24hCcr, Albumin, and Hemoglobin.** | | | | | |
| --- | --- | --- | --- | --- | --- |
| Variable | B | 95% CI | ꞵ | t | *P-value* |
| Hypothyroidism | 0.043 | -0.025 to 0.111 | 0.048 | 1.24 | 0.217 |
| Sex (male) | 0.177 | 0.155 to 0.199 | 0.466 | 15.80 | <0.001^**^ |
| Age | -0.003 | -0.004 to -0.001 | -0.099 | -3.16 | 0.002^**^ |
| BMI | 0.026 | 0.021 to 0.030 | 0.302 | 10.42 | <0.001^**^ |
| 24hrCcr | 0.005 | 0.005 to 0.006 | 0.543 | 15.71 | <0.001^**^ |
| Albumin | 0.048 | 0.011 to 0.085 | 0.081 | 2.56 | 0.011^*^ |
| Hemoglobin | -0.001 | -0.011 to 0.010 | -0.004 | -0.14 | 0.893 |
| constant | 0.048 |  |  |  | <0.001^**^ |
| **II: Adjusted for FT3, sex, age, BMI, 24hCcr, Albumin, and Hemoglobin.** | | | | | |
| Variable | B | 95% CI | ꞵ | t | *P-value* |
| FT3 | 0.030 | -0.014 to 0.073 | 0.045 | 1.34 | 0.181 |
| Sex (male) | 0.175 | 0.152 to 0.197 | 0.460 | 15.51 | <0.001^**^ |
| Age | -0.002 | -0.004 to -0.001 | -0.092 | -2.93 | 0.004^**^ |
| BMI | 0.025 | 0.020 to 0.030 | 0.298 | 10.21 | <0.001^**^ |
| 24hrCcr | 0.005 | 0.004 to 0.006 | 0.521 | 15.58 | <0.001^**^ |
| Albumin | 0.037 | -0.002 to 0.076 | 0.062 | 1.84 | 0.066 |
| Hemoglobin | -0.001 | -0.012 to 0.009 | -0.008 | -0.23 | 0.821 |
| constant | 0.008 |  |  |  | <0.001^**^ |

BMI, body mass index; 24hrCcr, 24-hour **creatinine** clearance; B, unstandardized regression coefficient; ꞵ, standardized coefficient; CI, confidence interval for B. Model R^2^ = 0.597, adjusted R^2^ = 0.590. *P <0.05, **P<0.01. FT3, free triiodothyronine; Model R^2^ = 0.596, adjusted R^2^ = 0.591. **P<0.01.
